# Development of a nomogram model for predicting pulmonary tuberculosis activity

**DOI:** 10.1097/MD.0000000000045582

**Published:** 2025-11-07

**Authors:** Xiaoyan Wu, Gonghui Wang, Hang Wang, Nana Li, Quanxian Liu

**Affiliations:** a Department of Tuberculosis, Affiliated Hospital of Zunyi Medical University, Zunyi, Guizhou Province, China.

**Keywords:** erythrocyte sedimentation rate, monocyte-to-HDL ratio, nomogram, predictive model, pulmonary tuberculosis, serum albumin

## Abstract

Timely and accurate identification of active pulmonary tuberculosis (APTB) is essential for effective treatment and public health control. This study aimed to develop a predictive nomogram using routine laboratory parameters to distinguish APTB from non-active pulmonary tuberculosis. A retrospective observational study was conducted at a single tertiary hospital from January 2021 to December 2024. A total of 356 newly diagnosed PTB patients were enrolled and classified into APTB (n = 225) or non-active pulmonary tuberculosis (n = 131) groups based on clinical, radiological, and microbiological criteria. Demographic, clinical, and laboratory data were collected. Univariate and multivariate logistic regression analyses were performed to identify independent predictors of APTB. A nomogram was constructed using 5 selected variables. Model performance was evaluated using receiver operating characteristic curves, calibration plots, and decision curve analysis. Multivariate analysis identified mean corpuscular volume, erythrocyte sedimentation rate, serum albumin, adenosine deaminase, and monocyte-to-high-density lipoprotein cholesterol ratio as independent predictors. The nomogram demonstrated strong discrimination (area under the curve = 0.913, sensitivity = 87.68%, specificity = 95.32%) and calibration (C-index = 0.915; Hosmer–Lemeshow *P* = .915). Decision curve analysis confirmed the model’s clinical utility. An internally validated nomogram incorporating 5 accessible laboratory indicators provides a reliable tool for predicting APTB, thereby facilitating timely diagnosis and supporting clinical decision-making.

## 1. Introduction

Pulmonary tuberculosis (PTB), caused by *Mycobacterium tuberculosis*, remains one of the most prevalent and deadly infectious diseases worldwide, particularly in low- and middle-income countries. Despite global public health efforts, tuberculosis continues to pose significant diagnostic and therapeutic challenges due to its heterogeneous clinical manifestations and the presence of latent or inactive disease states that can transition into active disease under certain conditions.^[1,2]^ According to the World Health Organization, an estimated 10.6 million individuals developed TB in 2022, with ~1.3 million TB-related deaths among human immunodeficiency virus (HIV)-negative individuals. Early and accurate identification of active PTB cases is essential not only for timely initiation of treatment but also for controlling disease transmission and improving clinical outcomes.^[3,4]^

Traditionally, the diagnosis of active PTB relies on a combination of clinical evaluation, radiographic findings, microbiological confirmation, and histopathological examination. However, these methods are often limited by prolonged turnaround time, low sensitivity in paucibacillary forms, and the requirement of high-level laboratory infrastructure, which may not be readily available in resource-constrained settings. Additionally, distinguishing active PTB from inactive or latent tuberculosis infection remains a complex task, especially in patients with atypical presentations or comorbid conditions. Given the limitations of conventional diagnostic tools, there is an urgent need for noninvasive, accurate, and practical predictive models to assist clinicians in assessing disease activity in suspected PTB cases.^[5,6]^ In recent years, predictive modeling has gained traction as a supplementary approach for clinical decision-making, particularly in infectious and chronic diseases. Nomograms, which provide a graphical representation of a statistical predictive model, have been increasingly applied in medical research to estimate individualized risk probabilities based on multivariate analysis. These models integrate multiple readily available clinical, laboratory, and radiological parameters into a single composite score, enabling personalized risk stratification and guiding diagnostic or therapeutic strategies.^[7]^

In this study, we aimed to construct and internally validate a nomogram based on clinical and laboratory parameters to predict PTB activity. By integrating routinely collected data, the model provides a practical and accessible tool for clinicians to estimate the likelihood of active PTB. This approach may facilitate earlier detection, timely initiation of therapy, and improved disease control in populations affected by tuberculosis.

## 2. Methods

### 2.1. Study design

This research was approved by the Ethics Committee of the Affiliated Hospital of Zunyi Medical University. This retrospective observational study was conducted to develop a predictive nomogram model for PTB activity. Patients diagnosed with PTB and managed at our institution between January 2021 and December 2024 were screened for eligibility. Individuals were included in the study if they met the following criteria: age ≥ 18 years at the time of diagnosis; clinically suspected or microbiologically or radiographically confirmed PTB, based on the diagnostic criteria established by the World Health Organization and the national tuberculosis control guidelines; availability of complete clinical data, including demographic information, clinical symptoms, laboratory indicators such as complete blood count, C-reactive protein (CRP), erythrocyte sedimentation rate (ESR), chest radiological findings (computed tomography or chest X-ray), and microbiological testing (including sputum smear microscopy, culture, or nucleic acid amplification tests [NAAT]); and no prior initiation of antituberculosis therapy at the time of data collection. Patients were excluded if they presented with any of the following conditions: isolated extrapulmonary tuberculosis without pulmonary involvement; confirmed co-infection with HIV, which may significantly affect host immune parameters and bias model performance; a history of malignancy, autoimmune diseases, or chronic inflammatory conditions (e.g., systemic lupus erythematosus, rheumatoid arthritis) known to interfere with routine inflammatory markers; or concurrent pulmonary infections of bacterial, viral, or fungal origin, as determined by clinical, laboratory, or imaging criteria.

After applying the inclusion and exclusion criteria, a total of 356 patients were enrolled in the study. Based on clinical, radiological, and microbiological evaluations, patients were categorized into an active pulmonary tuberculosis (APTB) group (n = 225) and a non-active pulmonary tuberculosis group (NAPTB) (n = 131). The study protocol was conducted in accordance with the ethical principles of the Declaration of Helsinki and was approved by the Institutional Review Board of our hospital. Informed consent was obtained from all participants prior to data collection.

### 2.2. Diagnostic criteria

Patients were classified into either the APTB group or the NAPTB group based on comprehensive clinical, radiological, microbiological, and histopathological assessments. All diagnoses were independently reviewed and confirmed by at least 2 experienced respiratory physicians or infectious disease specialists to ensure diagnostic consistency and accuracy.

Patients were assigned to the APTB group if they met any of the following criteria indicative of active disease:

1. Microbiological confirmation, including at least one of the following: Positive *Mycobacterium tuberculosis* culture from sputum or bronchoalveolar lavage fluid; positive NAAT for *M. tuberculosis* (GeneXpert MTB/RIF assay); positive acid-fast bacilli smear microscopy with clinical and radiological findings consistent with active PTB.
2. Clinical and radiographic evidence highly suggestive of active PTB, in the absence of microbiological confirmation, including: persistent cough, fever, night sweats, hemoptysis, or weight loss; imaging findings such as cavitary lesions, patchy infiltrates, or tree-in-bud patterns on chest X-ray or computed tomography; and favorable response to empirical antituberculosis therapy with subsequent clinical or radiological improvement.
3. Histopathological evidence from lung tissue biopsy showing granulomatous inflammation with caseous necrosis, consistent with active tuberculosis.

Patients were categorized into the NAPTB group if they met the following criteria:

1. History of previous pulmonary tuberculosis with evidence of stable fibrotic or calcified lesions on imaging, without clinical or microbiological indicators of active disease;
2. Absence of clinical symptoms consistent with active tuberculosis (e.g., no cough, fever, or weight loss);
3. Negative microbiological results, including acid-fast bacilli smear, culture, and NAAT;
4. Radiological imaging demonstrating stable, nonprogressive lesions such as fibrotic scarring, calcified nodules, or pleural thickening, without features indicative of active infection.

### 2.3. Data collection and outcome measures

Clinical and laboratory data were retrospectively collected from all eligible patients using electronic medical records. Collected variables included demographic information (age and sex), clinical presentations (including typical symptoms such as fever, cough, chest pain, and night sweats), and comorbid conditions (e.g., diabetes mellitus). Microbiological status was recorded based on bacteriological confirmation of *M. tuberculosis*. Hematological and biochemical parameters included white blood cell count, percentage of neutrophils, platelet count, platelet-to-lymphocyte ratio (PLR), neutrophil-to-lymphocyte ratio (NLR), high-sensitivity C-reactive protein (hs-CRP), hs-CRP-to-albumin ratio, hs-CRP-to-prealbumin, hs-CRP-to-lymphocyte ratio, ESR, monocyte-to-high-density lipoprotein cholesterol ratio (MHR), procalcitonin, lactate dehydrogenase (LDH), alkaline phosphatase, adenosine deaminase (ADA), mean corpuscular volume (MCV), hematocrit, hemoglobin, mean corpuscular hemoglobin concentration, mean platelet volume, platelet distribution width, blood urea nitrogen, serum sodium, LDH-to-adenosine deaminase ratio, and serum albumin. All laboratory tests were performed within 24 hours of patient admission to ensure the consistency and comparability of baseline measurements.

### 2.4. Statistical analysis

All statistical analyses were conducted using R software (version 4.3.1) and SPSS (version 28.0; IBM Corp., Armonk). Continuous variables were presented as mean ± standard deviation or median with interquartile range, depending on the normality of distribution assessed by the Shapiro–Wilk test. Categorical variables were summarized as frequencies and percentages. Differences between the APTB group and the non-active group were evaluated using the independent-samples *t* test or Mann–Whitney *U* test for continuous variables and the chi-square test or Fisher exact test for categorical variables, as appropriate. Univariate logistic regression analysis was performed to screen for variables associated with PTB activity. Variables with a *P*-value < .10 were included in the multivariate logistic regression model using a stepwise backward elimination approach based on the Akaike information criterion. Adjusted odds ratios (ORs) with 95% confidence intervals were reported to reflect the magnitude of association.

A nomogram was subsequently constructed based on the final multivariate logistic regression model using the “rms” package in R. Model discrimination was assessed by calculating the concordance index (C-index) and the area under the receiver operating characteristic curve (AUC). Calibration of the nomogram was evaluated through calibration plots generated using 1000-bootstrap resamples to compare predicted probabilities with observed outcomes. In addition, decision curve analysis (DCA) was performed to determine the net clinical benefit of the model across a range of threshold probabilities. All statistical tests were 2-sided, and a *P*-value < .05 was considered statistically significant. For variables with missing data, multiple imputations were performed using the chained equations method (MICE). Ten imputed datasets were generated, incorporating all candidate predictor variables and outcome status to preserve associations among variables. The imputed datasets were then analyzed separately, and the results were pooled according to Rubin rules under the assumption that data were missing at random.

## 3. Results

### 3.1. Comparison of baseline characteristics between APTB and NAPTB groups

Baseline clinical and laboratory characteristics differed significantly between the APTB and NAPTB groups. No significant differences were observed in sex distribution or age. However, typical tuberculosis symptoms and comorbid diabetes were more frequently reported in the APTB group (*P* < .05). Laboratory parameters indicated that patients with APTB had significantly elevated markers of systemic inflammation, including white blood cell, percentage of neutrophils, platelet count, ESR, hs-CRP, and derived ratios such as PLR, NLR, hs-CRP-to-albumin ratio, hs-CRP-to-prealbumin ratio, hs-CRP-to-lymphocyte ratio, and MHR (all *P* < .001). In contrast, the APTB group showed lower levels of nutritional and hematologic indicators, including MCV, hemoglobin, hematocrit, mean corpuscular hemoglobin concentration, albumin, and sodium (*P* < .001). Additionally, markers related to tissue injury and immune activation, such as procalcitonin, LDH, alkaline phosphatase, ADA, and LDH-to-adenosine deaminase ratio, were significantly increased in the APTB group (*P* < .001) (Table 1).

**Table 1:** Comparison of baseline clinical and laboratory characteristics between active and non-active pulmonary tuberculosis groups.

Variable	NAPTB group (n = 131)	APTB group (n = 225)	χ²/t	*P*-value
Sex (male/female)	80/51	129/96	0.477	.49
Typical symptoms [%]	66 (50.4%)	186 (82.7%)	41.731	<.001
Diabetes [%]	9 (6.9%)	36 (16.0%)	6.25	.012
Age (yr)	45.3 ± 6.1	46.1 ± 5.6	0.314	.753
WBC (×10⁹/L)	12.7 ± 4.4	14.9 ± 4.2	4.658	<.001
N%	71.8 ± 3.9	76.2 ± 4.6	4.957	<.001
PLT (×10⁹/L)	214.1 ± 53.4	255.8 ± 47.3	6.659	<.001
MCV (fl)	96.1 ± 5.8	87.2 ± 3.6	5.024	<.001
HCT	0.41 ± 0.10	0.39 ± 0.10	4.457	<.001
Hb (g/L)	120.3 ± 9.5	114.2 ± 8.1	4.232	<.001
MCHC (g/L)	321.4 ± 25.6	306.5 ± 15.9	8.625	<.001
MPV (fl)	9.4 ± 1.3	7.4 ± 0.9	5.236	<.001
PDW (%)	17.0 ± 2.5	14.8 ± 1.3	4.085	<.001
PLR	135.9 ± 29.6	161.2 ± 33.0	21.256	<.001
NLR	2.4 ± 0.8	4.4 ± 1.2	10.203	<.001
hs-CRP (mg/L)	12.6 ± 6.7	24.1 ± 10.5	19.658	<.001
HSCAR	0.31 ± 0.10	0.68 ± 0.21	16.624	<.001
HSCPR	0.42 ± 0.12	0.81 ± 0.29	20.214	<.001
HSCLR	8.6 ± 2.8	15.0 ± 4.4	14.627	<.001
ESR (mm/hr)	25.0 ± 6.7	29.9 ± 7.4	12.325	<.001
MHR	0.29 ± 0.09	0.42 ± 0.10	5.096	<.001
PCT (ng/L)	1.94 ± 0.31	5.72 ± 2.18	16.627	<.001
LDH (U/L)	122.0 ± 31.0	160.3 ± 34.2	22.528	<.001
ALP (U/L)	160.5 ± 33.0	189.1 ± 29.3	26.674	<.001
ADA (U/L)	10.8 ± 3.2	17.9 ± 4.3	7.859	<.001
BUN (mg/L)	8.0 ± 1.6	6.4 ± 1.4	3.629	<.001
Sodium (mmol/L)	124.3 ± 4.7	121.0 ± 3.7	3.967	<.001
LAR	0.49 ± 0.11	0.42 ± 0.09	4.127	<.001
Albumin (g/L)	41.2 ± 5.8	32.1 ± 3.4	9.658	<.001

ADA = adenosine deaminase, ALP = alkaline phosphatase, APTB = Active Pulmonary Tuberculosis, BUN = blood urea nitrogen, ESR = erythrocyte sedimentation rate, Hb = hemoglobin, HCT = hematocrit, HSCAR = hs-CRP-to-albumin ratio, HSCLR = hs-CRP-to-lymphocyte ratio, HSCPR = hs-CRP-to-prealbumin ratio, hs-CRP = high-sensitivity C-reactive protein, LAR = lactate dehydrogenase-to-adenosine deaminase ratio, LDH = lactate dehydrogenase, MCHC = mean corpuscular hemoglobin concentration, MCV = mean corpuscular volume, MHR = monocyte-to-high-density lipoprotein cholesterol ratio, MPV = mean platelet volume, N% = percentage of neutrophils, NAPTB = non-active pulmonary tuberculosis, NLR = neutrophil-to-lymphocyte ratio, PCT = procalcitonin, PDW = platelet volume distribution width, PLR = platelet-to-lymphocyte ratio, PLT = platelet count, WBC = white blood cell count.

### 3.2. Multivariate logistic regression analysis of risk factors for APTB

Multivariate logistic regression identified 5 independent predictors of APTB. Reduced MCV and low serum albumin were the strongest predictors, with ORs of 2.474 and 2.939, respectively (*P* < .001). Elevated ESR, ADA, and MHR were also significantly associated with increased risk of APTB (all *P* < .001). These findings suggest that hematologic, inflammatory, and metabolic markers may contribute to distinguishing active from NAPTB (Table 2).

**Table 2:** Multivariate logistic regression analysis of predictive factors for active pulmonary.

Factor	Regression coefficient	Standard error value	Wald value	OR	95% CI	*P*-value
MCV	0.906	0.275	10.854	2.474	1.443–4.241	<.001
ESR	0.521	0.390	1.785	1.684	1.184–3.617	<.001
Albumin	1.078	0.346	9.707	2.939	1.492–5.791	<.001
ADA	0.879	0.320	7.545	2.409	1.287–4.511	<.001
MHR	0.747	0.231	10.457	2.111	1.342–3.320	<.001
HSCLR	1.525	0.506	9.061	4.596	2.358–9.358	<.001

Tuberculosis.

ADA = adenosine deaminase, ESR = erythrocyte sedimentation rate, HSCLR = high-sensitivity C-reactive protein to lymphocyte ratio, MCV = mean corpuscular volume, MHR = monocyte-to-high-density lipoprotein cholesterol ratio, OR = odds ratio.

### 3.3. Construction and validation of the nomogram model for predicting active pulmonary tuberculosis

**Figure 1.**
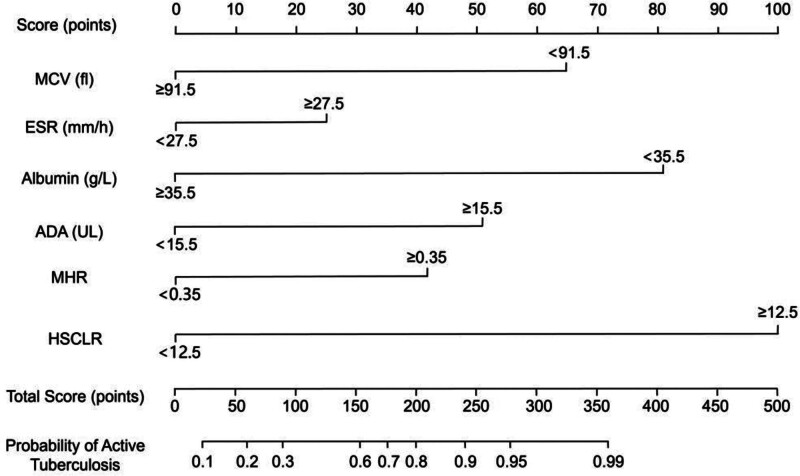
Nomogram for predicting the probability of active pulmonary tuberculosis. Each predictor (MCV, ESR, albumin, ADA, and MHR) is assigned a point value based on its regression coefficient. The total score is calculated by summing all individual points, which corresponds to a predicted probability of active pulmonary tuberculosis (APTB) at the bottom scale. MCV = mean corpuscular volume, ESR = erythrocyte sedimentation rate, ADA = adenosine deaminase, MHR = monocyte-to-high-density lipoprotein cholesterol ratio.

Based on the 5 independent predictors identified by multivariate logistic regression analysis, MCV, ESR, serum albumin, ADA, and MHR, a nomogram model was constructed to estimate the individual probability of APTB. Each predictor was assigned a specific point value according to its regression coefficient. The total score was calculated by summing the individual points, with higher scores corresponding to a greater risk of APTB (Fig. 1).

### 3.4. Discrimination performance of the nomogram

**Figure 2.**
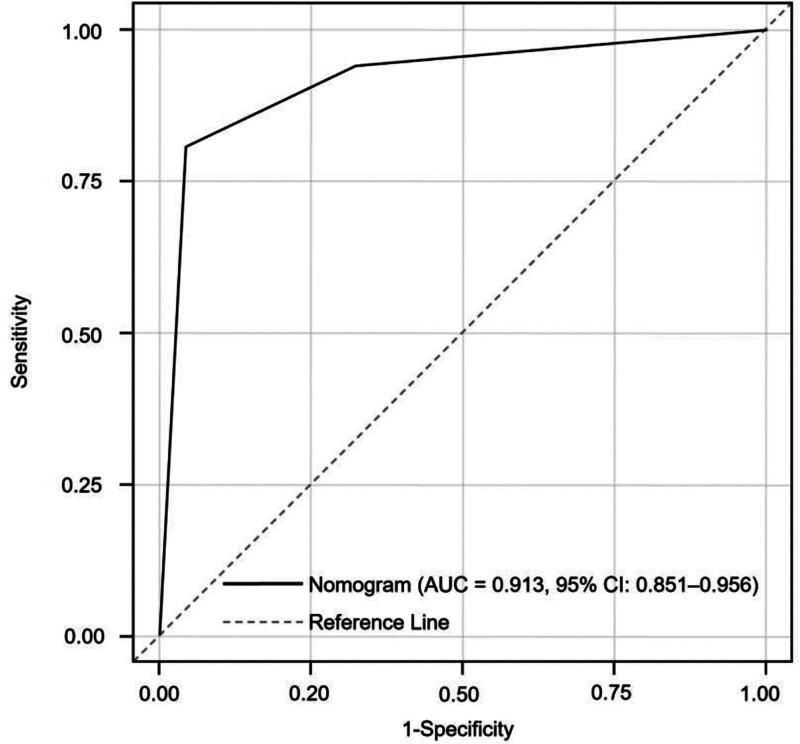
Receiver operating characteristic (ROC) curve of the nomogram for predicting APTB. The area under the ROC curve (AUC) was 0.913 (95% CI: 0.851–0.956), indicating excellent discrimination between active and non-active pulmonary tuberculosis cases. APTB = active pulmonary tuberculosis, CI = confidence interval.

The discriminative ability of the nomogram was assessed by plotting the receiver operating characteristic curve and calculating the AUC. The model demonstrated excellent discrimination, with an AUC of 0.913 (95% CI: 0.851–0.956). When the optimal cutoff point was determined by the Youden index, the model achieved a sensitivity of 87.68% and a specificity of 95.32% (Fig. 2), indicating high accuracy in distinguishing APTB from non-active cases.

### 3.5. Calibration of the nomogram

**Figure 3.**
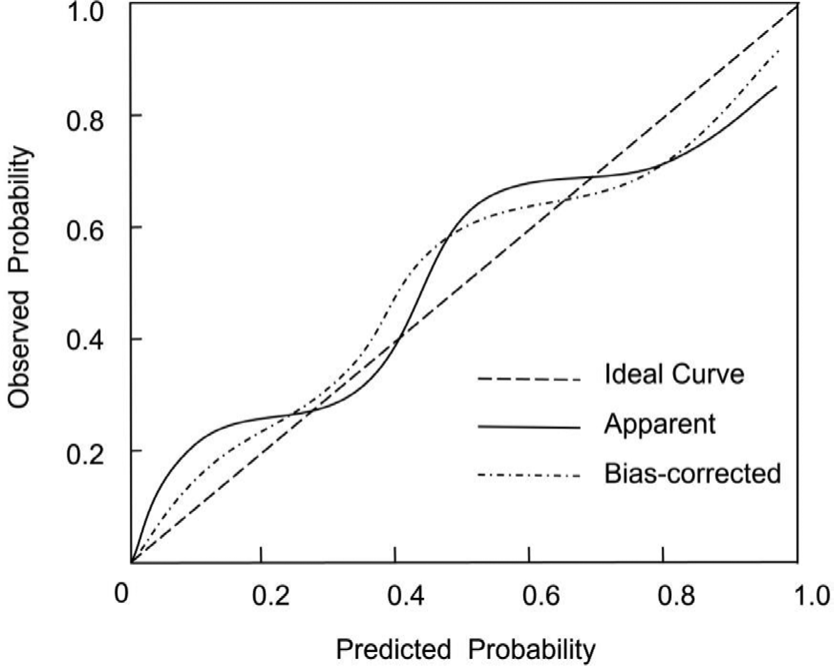
Calibration curve of the nomogram model based on 1000-bootstrap resamples. The calibration curve shows good agreement between predicted probabilities and observed outcomes.

Internal validation was performed using 1000-bootstrap resamples. The C-index was 0.915 (95% CI: 0.865–0.961), suggesting excellent model stability and discrimination. Calibration was further evaluated using the Hosmer–Lemeshow goodness-of-fit test, yielding a nonsignificant result (χ² = 2.569, *P* = .915), indicating good agreement between predicted and observed outcomes. The calibration curve also demonstrated strong concordance, supporting the model’s reliability in risk estimation (Fig. 3).

### 3.6. Clinical utility of the nomogram

**Figure 4.**
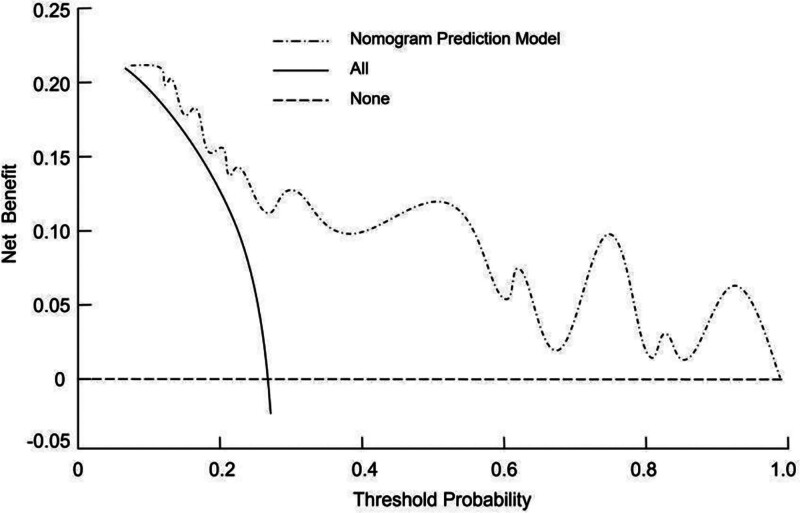
Decision curve analysis (DCA) for the nomogram predicting APTB. The DCA curve demonstrates that the nomogram provides a higher net clinical benefit across a range of threshold probabilities compared with the default strategies of treating all patients or none, indicating its potential utility in clinical decision-making. APTB = active pulmonary tuberculosis.

DCA was employed to evaluate the clinical benefit of the nomogram. The decision curve showed that the model provided a higher net benefit across a range of threshold probabilities compared with the default strategies of treating all or no patients. This indicates that the nomogram may offer meaningful clinical utility in guiding individualized decision-making for APTB screening and diagnosis (Fig. 4).

## 4. Discussion

The present study developed and internally validated a novel nomogram model for predicting APTB based on 5 routine clinical variables: MCV, ESR, ADA, and MHR. This model demonstrated high diagnostic accuracy, with an AUC of 0.913, sensitivity of 87.68%, and specificity of 95.32%, underscoring its potential utility in early APTB identification among hospitalized patients. From a pathophysiological perspective, the selection of predictors in our nomogram is supported by existing evidence and reflects the multifactorial nature of TB disease. MCV, a marker of erythrocyte size, is often decreased in the context of chronic inflammatory diseases such as TB due to iron-restricted erythropoiesis. Inflammatory cytokines in TB inhibit erythropoietin response and iron utilization, leading to microcytic anemia. Our findings that lower MCV independently predicted APTB are consistent with prior studies linking microcytosis with TB-associated anemia and systemic inflammation.^[8,9]^

Albumin, a negative acute-phase reactant, was another strong predictor. Hypoalbuminemia in TB is common and reflects both malnutrition and systemic inflammation. Low serum albumin levels have been associated with delayed sputum conversion, extensive pulmonary lesions, and worse clinical outcomes. The predictive power of albumin (OR ~ 2.94) in our model reaffirms its value as a reliable surrogate for host nutritional status and disease burden. Notably, albumin is widely available and inexpensive, making it a practical parameter in resource-limited settings. ESR, a nonspecific marker of inflammation, was also independently associated with APTB.^[10,11]^ Elevated ESR levels result from increased fibrinogen and globulin concentrations during inflammation, both of which are commonly seen in active TB. Although ESR lacks specificity, its elevation in active disease has long been recognized. In our analysis, ESR remained a significant factor even when controlling for other inflammatory markers, highlighting its retained diagnostic utility. ADA, an enzyme involved in purine metabolism, reflects T-cell activation.^[12]^ Elevated serum ADA has been traditionally used for diagnosing TB pleuritis, but recent evidence also supports its role in active pulmonary TB. Increased ADA levels indicate heightened cell-mediated immune responses, which are characteristic of active TB. The inclusion of ADA in our nomogram offers an immune-related dimension to the predictive model and aligns with previous studies that reported ADA as a valuable diagnostic adjunct, particularly when microbiological evidence is inconclusive.^[13,14]^

MHR is a novel marker of systemic inflammation and metabolic dysregulation, calculated by dividing monocyte count by HDL cholesterol level. In TB, monocyte counts typically rise due to chronic immune activation, while HDL decreases as part of the inflammatory response. High MHR thus signifies a pro-inflammatory and immunometabolic milieu.^[15]^ Although less frequently studied in TB compared with markers such as NLR or PLR, MHR has gained recognition in cardiovascular and infectious disease research as a predictor of severity and outcomes. Our study is among the first to demonstrate its independent association with APTB, suggesting its potential relevance in TB risk stratification.^[16,17]^ A key strength of this study lies in the simplicity and accessibility of the included variables. All 5 predictors are part of routine laboratory panels, allowing the nomogram to be readily implemented in most healthcare settings without additional testing costs. This contrasts with other TB prediction models that rely on advanced immunologic assays (e.g., interferon-gamma release assays) or imaging techniques, which may be less feasible in low-resource environments. Our model thus addresses a critical gap in TB diagnostics by offering a practical tool suitable for use in both tertiary and peripheral centers.^[18,19]^

The nomogram’s performance was robust, as evidenced by the high AUC, C-index, and favorable results of the Hosmer–Lemeshow test. Internal validation via 1000-bootstrap resampling confirmed its reliability and minimized the risk of overfitting. DCA further indicated that the model provides a net clinical benefit across a range of threshold probabilities, supporting its use in guiding clinical decision-making. Clinically, the model can assist in early risk assessment of patients presenting with suspected pulmonary TB. For instance, individuals with elevated ESR and ADA, reduced albumin and MCV, and high MHR may be prioritized for confirmatory testing (e.g., sputum culture, GeneXpert) or early treatment initiation. This is particularly valuable in cases with ambiguous presentations, smear-negative results, or where diagnostic delays pose a risk to patient outcomes and public health.

In comparison with existing diagnostic tools, our nomogram provides certain advantages and also has limitations. Molecular assays such as Xpert MTB/RIF are widely used and highly specific for detecting *M. tuberculosis* and rifampicin resistance; however, their sensitivity is reduced in smear-negative or paucibacillary cases, and they may be unavailable in resource-limited settings due to cost and infrastructure requirements. Imaging-based prediction models and radiomics nomograms have also been proposed, but these approaches often rely on high-quality imaging data and advanced analytical platforms, which may limit their generalizability. Compared with these modalities, our nomogram is constructed from 5 routinely available laboratory parameters, making it simple, inexpensive, and broadly applicable in most clinical laboratories. While our model is not intended to replace microbiological or molecular confirmation, it may serve as a complementary tool in the clinical pathway, particularly for early risk stratification, prioritizing patients for confirmatory testing, and assisting decision-making when diagnostic uncertainty exists. Future studies directly comparing our model with established diagnostic tools will be essential to better define its relative performance and practical feasibility in different healthcare contexts.

Despite its strengths, this study has several limitations. First, it was a single-center, retrospective analysis of hospitalized, newly diagnosed TB patients. The absence of external or independent validation cohorts restricts the generalizability and clinical applicability of the nomogram to broader populations and different healthcare settings. Future studies incorporating multicenter, prospective cohorts with external validation are essential to confirm the robustness of the model and facilitate its clinical translation. Second, some predictors such as ADA and HDL (for MHR) may not be routinely tested in all facilities, though they are generally affordable and available. Third, the model is diagnostic, not prognostic; it does not assess treatment outcomes or recurrence risk. HIV status was also not included, which may affect model performance in immunocompromised patients. Additionally, our model was not directly compared with other diagnostic tools such as imaging or molecular tests. Lastly, because of the distinct clinical separation between APTB and NAPTB in our cohort, there is a risk of spectrum bias, which may lead to the overestimation of model performance in real-world practice. Furthermore, unmeasured or uncontrolled confounding factors, such as nutritional status, HIV infection, or other comorbidities, could have influenced biomarker levels and model discrimination. These issues highlight the need for future prospective, multicenter studies that incorporate broader patient populations and carefully account for potential confounders.

## 5. Conclusions

In conclusion, this study developed an internally validated nomogram based on 5 routine laboratory indicators, MCV, ESR, albumin, ADA, and MHR, for the accurate prediction of APTB. The model demonstrated excellent discrimination and calibration, providing a practical and low-cost tool to facilitate early diagnosis and risk stratification in clinical settings.

## Author contributions

**Conceptualization:** Xiaoyan Wu, Gonghui Wang, Hang Wang, Quanxian Liu.

**Data curation:** Xiaoyan Wu, Gonghui Wang, Hang Wang, Quanxian Liu.

**Formal analysis:** Xiaoyan Wu, Quanxian Liu.

**Investigation:** Xiaoyan Wu, Gonghui Wang, Hang Wang, Nana Li, Quanxian Liu.

**Methodology:** Xiaoyan Wu, Hang Wang, Nana Li, Quanxian Liu.

**Supervision:** Xiaoyan Wu, Gonghui Wang, Hang Wang, Quanxian Liu.

**Validation:** Xiaoyan Wu, Gonghui Wang, Quanxian Liu.

**Visualization:** Xiaoyan Wu, Gonghui Wang, Nana Li, Quanxian Liu.

**Writing – original draft:** Xiaoyan Wu, Quanxian Liu.

**Writing – review & editing:** Xiaoyan Wu, Quanxian Liu.
